# Signature of gene expression profile of liver sinusoidal endothelial cells in nonalcoholic steatohepatitis

**DOI:** 10.3389/fcell.2022.946566

**Published:** 2022-09-21

**Authors:** Yang Wang, Yifan Zhang, Yimin Li, Yun Liu, Yulan Liu

**Affiliations:** ^1^ Department of Gastroenterology, Peking University People’s Hospital, Beijing, China; ^2^ Clinical Center of Immune-Mediated Digestive Diseases, Peking University People’s Hospital, Beijing, China; ^3^ Department of Rheumatology and Immunology, Beijing Key Laboratory for Rheumatism Mechanism and Immune Diagnosis, Peking University People’s Hospital, Beijing, China

**Keywords:** liver sinusoidal endothelial cells, nonalcoholic steatohepatitis, gene expression, inflammation, miRNA, lncRNA, circRNA

## Abstract

**Background:** There has been emerging evidence that liver sinusoidal endothelial cells (LSECs) play an important role in the pathogenesis of nonalcoholic steatohepatitis (NASH). This study aims to figure out the signature of the gene expression profile of LSECs in NASH and to explore potential biomarkers related to damaged LSECs in NASH.

**Methods and materials:** Animal experiments were performed to demonstrate the significant structural damage of LSECs in the NASH model. To further understand the functional changes of these damaged LSECs in NASH, we used the public GEO database that contained microarray data for the gene expression of LSECs in NASH and normal mouse liver. Differentially expressed genes (DEGs) were analyzed, and further Gene Ontology (GO) enrichment analysis was performed to understand the functional changes. The hub genes were then identified and validated *via* external GEO databases.

**Results:** There was significant structural damage to LSECs in the NASH model, accompanied by remarkable functional changes of LSECs with 174 DEGs (156 upregulated and 18 downregulated genes). The functions of these DEGs were mainly enriched in the inflammatory reactions and immune responses. Nine specifically expressed hub genes were identified. Among them, CCL4 and ITGAX showed the most significant correlation with NASH, with AUROC of 0.77 and 0.86, respectively. The protein–protein interaction network, mRNA–miRNA interaction network, and ceRNA network were further predicted.

**Conclusion:** LSECs show significant structural damage and functional changes in NASH. The LSEC-related DEGs, such as CCL4 and ITGAX, might be promising biomarkers as well as potential treatment targets for NASH.

## Introduction

Nonalcoholic steatohepatitis (NASH) has become a leading cause of cirrhosis and hepatocellular carcinoma over recent years with increased overall mortality (Dufour et al., 2020). The ballooning degeneration of hepatocytes and lobular inflammation are the histological characteristics of disease progression from nonalcoholic fatty liver disease (NAFLD) to NASH (Kleiner et al., 2019). Early diagnosis of NASH is of critical importance to reduce the economic burden for patients with NASH. However, the liver pathological biopsy, the gold standard for diagnosing NASH, is still difficult to obtain for most patients.

The liver contains a large number of hepatocytes and multiple types of non-parenchymal cells, including liver sinusoidal endothelial cells (LSECs), Kupffer cells, natural killer (NK) cells, B lymphocytes, T lymphocytes, and hepatic stellate cells. Recent reports have brought the importance of LSECs to our attention as the role of the hepatic barrier against invasive factors (Wang and Liu, 2021a; Wang et al., 2021). However, under pathological conditions, LSECs can shift to a pro-inflammatory phenotype and thus contributing to NASH progression (Hammoutene and Rautou, 2019). LSECs are not considered innate immune cells, but they might show immunomodulatory functions under stressed conditions (Cai et al., 2019). For instance, in many types of liver diseases, LSECs can mediate the adhesion of circulating leukocytes (Carambia et al., 2014; Wang and Liu, 2021b). The damage of LSECs has been considered important for the activation of immune reactions in the progression of NASH. Injured LSECs show both structural and functional changes. For instance, in congestive hepatopathy, the mechanical stretch of LSECs could increase the expression of CXCL1 *via* integrin-dependent activation of transcription factors (Hilscher et al., 2019). LSECs can also release mechanosensitive angiocrine signals that further promote portal hypertension by recruiting sinusoidal neutrophils and promoting the formation of neutrophil extracellular traps and microthrombi (Hilscher et al., 2019). Under the stimulation of lipopolysaccharide (LPS), injured LSECs can express significantly increased chemokines and inflammatory factors and further mediate the recruitment of hepatic neutrophils to accelerate liver injury (Ng-Nguyen et al., 2021). The exact mechanisms of injured LSECs in inducing hepatic inflammation and liver diseases remain to be further investigated.

Currently, transcriptomic and microarray analysis has been widely applied to explore new biomarkers in a variety of diseases including cancers (Krishnan et al., 2021), metabolic diseases (Fujimoto et al., 2020), and auto-immune diseases (Cheng et al., 2021). In addition, verification of the competitive endogenous RNA (ceRNA) networks can present a deeper mechanism in the transcriptional regulatory network (Demircioglu et al., 2019). While a remarkable change in transcription is recognized as a defining feature of NASH, the potential key genes and pathway networks of LSECs are worth thorough analysis. Thus, in this study, we aim to comprehensively analyze the signature of the gene expression profile of LSECs in NASH.

Our study suggests great changes in the gene expression profile of LSECs in NASH and also predicts new biomarkers and pathways to further explore the interplay of regulatory mechanisms.

## Methods

### Animal experiments

Animal experiments were performed using 250–300 g male Sprague–Dawley rats, which were obtained from Beijing Vital River Laboratory Animal Technology Co., Ltd. The rats were fed with the methionine–choline-deficient diet (MCD) (obtained from Jiangsu Medicine Co., Ltd) for 6 weeks to induce NASH, while a normal chow diet was used for the control group. Rats were kept in cages in a temperature-controlled room (21 ± 2°C) with free access to food and water. Animal experiments were approved and supervised by the Ethics Committee of Peking University People’s Hospital (Approval no. 2020PHE074).

### Measurement of alanine aminotransferase (ALT) and aspartate aminotransferase (AST)

At the appropriate time point of disease modeling, rats were euthanized by CO_2_ asphyxiation, and blood samples were collected. After centrifugation of blood samples for 10 min at 3,200 rpm, serum was collected. ALT and AST were tested to evaluate liver injury by the assay reagent kits (Nanjing Jiancheng Bioengineering Institute, China) according to the manufacturer's instructions.

### H and E staining

Liver tissue was fixed in paraformaldehyde and embedded in paraffin. The liver sample was cut into 4 μm thick sections and then baked at 60°C for 4 h and embedded in paraffin. After paraffin was removed by a graded ethanol series and xylene, sections were stained with hematoxylin and eosin.

### Scanning electron microscopy (SEM) and transmission electron microscopy (TEM)

The perfusion fixation of rat liver tissue for light and electron microscopy was performed as previously described (Wisse et al., 1984). Briefly, under isoflurane anesthesia, the liver of SD rats was perfused to scour blood, followed by glutaraldehyde fixative buffer *via* the portal vein. The perfusion was stopped until the surface of the liver changed from red and soft to pale and tough. Liver samples were cut into 2 × 2 × 2 mm SEM (JSM-7900F, JEOL) and appropriate slices for TEM.

### Microarray data acquisition

The public GEO database (https://www.ncbi.nlm.nih.gov/gds/) was searched to obtain microarray data for LSECs in NASH and normal mouse liver. The screening criteria included (1) expression profiling that was tested by array (2); LSECs were isolated from NASH and normal samples from liver biopsies (3); datasets contained no less than five samples (4); datasets contained full-scale information about the samples (5); one biopsy sample of each subject was analyzed without replicates. Finally, only one GPL24557 dataset, GSE140994 (Winkler et al., 2021), which included five normal samples and five NASH samples, was selected as the test set. In addition, six datasets including GSE17470 (Baker et al., 2010), GSE63067 (Frades et al., 2015), GSE35961 (Kita et al., 2012), GSE93819 (Kobori et al., 2017), GSE59492 (Blaya et al., 2016), and GSE33857 (Murakami et al., 2012) were selected as the validation sets (Table 1).

**TABLE 1:** Information of selected GEO datasets.

GEO accession	Platform	Study type	Sample type	No. of samples		Attribute
				Case	Control	
GSE140994	GPL24557	Expression profiling by array	LSEC from NASH/NC mouse liver	5	5	Test set
GSE17470	GPL2895	Expression profiling by array	Liver from NASH patients or control	7	4	Validation set
GSE63067	GPL570	Expression profiling by array	Liver from NASH patients or control	9	7	Validation set
GSE35961	GPL1261	Expression profiling by array	Liver from NASH mice or control	4	4	Validation set
GSE93819	GPL1261	Expression profiling by array	Liver from NASH mice or control	5	5	Validation set
GSE59492	GPL16384	Non-coding RNA profiling by array	Liver from NASH patients or control	5	6	Validation set
GSE33857	GPL10656	Non-coding RNA profiling by array	Serum from NASH patients or control	7	12	Validation set

Abbreviation: GEO, Gene Expression Omnibus; LSEC, liver sinusoidal endothelial cell; NC, normal control.

### Data normalization and identification of differentially expressed genes (DEGs)

The original files that were downloaded from the GEO databases were preprocessed and normalized by the robust multiarray average (RMA) method by using the GEOquery package in R software (version 4.0.1). The limma R package was used to conduct gene analysis to identify the DEGs of intergroup differences *via* the “makeContrasts” code. The screening criteria were log2 (fold change) > 2 or < -2 with an adjusted *p*-value (Q value) < 0.05.

### Analysis for heatmap and volcano plot

We performed principal component analysis and drew heatmaps and volcano plots to visualize these DEGs by using the ggplot function and pheatmap package in R software. The online tool BioGPS (http://biogps.org/) was used to assess the tissue-/organ-specific expressed genes. The screening criteria of tissue-specific genes were as follows (1) transcripts that mapped to a specific organ system with an expression value of >10 multiples of the median and (2) second most abundant tissue’s expression were no more than half as high.

### Enrichment analysis by GO database

We analyzed the biological process and molecular function according to Gene Ontology (GO) classification to uncover the functions of DEGs and to further predict the biological connections in these genes. The gene list consisting of differentially expressed mRNAs screened was submitted to the DAVID database (https://david.ncifcrf.gov/home.jsp), which is a web-based bioinformatics resource for functional enrichment analysis. The R software cluster profiler package was used for the visualization of the GO enrichment. The top ten GO terms with the most significant *p* values were selected according to the biological process and molecular function analysis.

### Prediction of the PPI network

A protein–protein interaction (PPI) network was predicted based on the DEGs to further explore the functional role of these DEGs by using the online STRING tool (https://strin-g-db.org/) with a filter condition (combined score >0.4) (Szklarczyk et al., 2021). Next, we analyzed the interaction information and drew the PPI network by using the plugin CytoHubba. Minimal Common Oncology Data Elements (MCODE) was used to identify three significant gene clusters and to calculate the cluster scores. Five algorithms, including degree, maximal clique centrality (MCC), maximum neighborhood component (MNC), density of maximum neighborhood component (DMNC), and clustering co-efficient, were utilized to filter the modules of core genes and to obtain the top 15 hub genes. CytoHubba was used to further identify crucial genes in this PPI network as hub genes. The final hub genes were then obtained based on the results of organ-/tissue-specific expression.

### Verification of specifically expressed hub genes by four datasets from the GEO database

GSE17470 and GSE63067, which included 16 liver samples from NASH patients and 11 liver samples from control patients, and GSE35961 and GSE93819, which included nine liver samples from NASH mice and nine liver samples from normal mice, were used to verify the specifically expressed hub genes. GraphPad Prism was used to analyze the expression profiles and ROC curves of specifically expressed hub genes.

### Prediction of mRNA–miRNA interaction network

We uploaded the specifically expressed hub genes to Network Analyst (http://www.networkanalyst.ca) to explore the potential miRNA–protein interactions. Based on the miRNA target gene prediction, Cytoscape software was used to plot the mRNA–miRNA interaction network and pathway enrichment analysis.

### Prediction of ceRNA network

StarBase version 3.0 (http://starbase.sysu.edu.cn/index.php) was used to predict the interaction of lncRNAs and circRNAs with miRNAs (Li et al., 2014). The intersections of the predicted results were used as the target lncRNAs and circRNAs. The ceRNA networks containing lncRNA, miRNA, and mRNA were visualized by the Cytoscape software.

### Statistical analysis

Student’s t-test was used to compare the differences between the two groups by IBM SPSS Statistics 25 (SPSS, Inc., Chicago, United States ). All the tests were two-tailed, and *p*-value less than 0.05 was considered statistically significant. GraphPad Prism 9 was used to perform diagnostic analysis and draw the ROC curves.

## Results

### Significant damage of LSECs in MCD diet-induced NASH model

**FIGURE 1:**
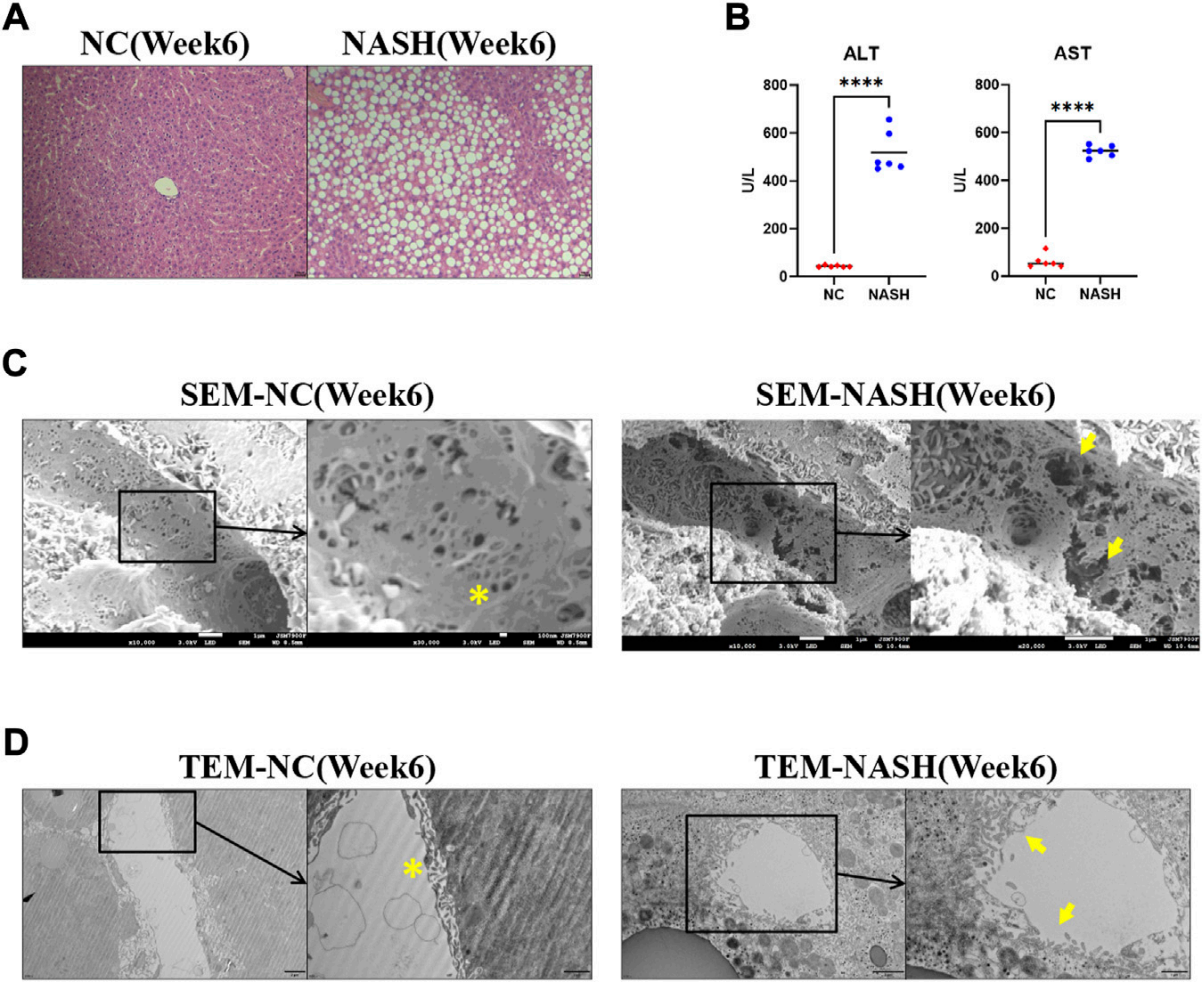
LSECs injury in the NASH model. **(A)** H&E staining was used to evaluate the pathology of the liver in two groups. **(B)** Alanine aminotransferase (ALT) and aspartate aminotransferase (AST) were tested to evaluate the liver injury. **(C,D)** Scanning electron microscopy (SEM) and transmission electron microscopy (TEM) showed the ultrastructural features of liver sinusoidal endothelial cells (LSECs) in the normal liver and NASH model. ns, non-significant; ****p < 0.001 by *t*-test; n = 6 at each time point per group; figures are representative of three experiments.

Wild-type rats were fed with an MCD diet for 6 weeks to induce NASH. H&E revealed steatosis, ballooning, inflammation, and fibrosis in the livers of MCD-fed rats (Figure 1A). NASH was associated with a remarkable increase in ALT and AST (Figure 1B). Normal LSECs on SEM and TEM imaging were characterized by regularly organized fenestrae and sieve plates on the sinusoidal endothelium (Figures 1C, D). While in the liver of the NASH model, remarkable formation of large defects on sinusoidal endothelium and LSEC lining detachment/deficiency were observed, indicating significant LSEC structural damage (Figures 1C, D).

### Identification of DEGs in damaged LSECs *via* GEO database

As shown in Table 1, a total of seven GEO databases were identified. The dataset GSE140994 contains the gene expression data of LSECs isolated from NASH and NC mouse livers and thus was used as the test set. The datasets, including GSE17470, GSE63067, GSE35961, and GSE93819, contain gene expression profiling of the liver from NASH patients or mice and thus were used as validation sets. GSE59492 and GSE33857 contain non-coding RNA profiling of the liver and serum from NASH patients, respectively, and were used to validate the miRNA expression profile.

**FIGURE 2:**
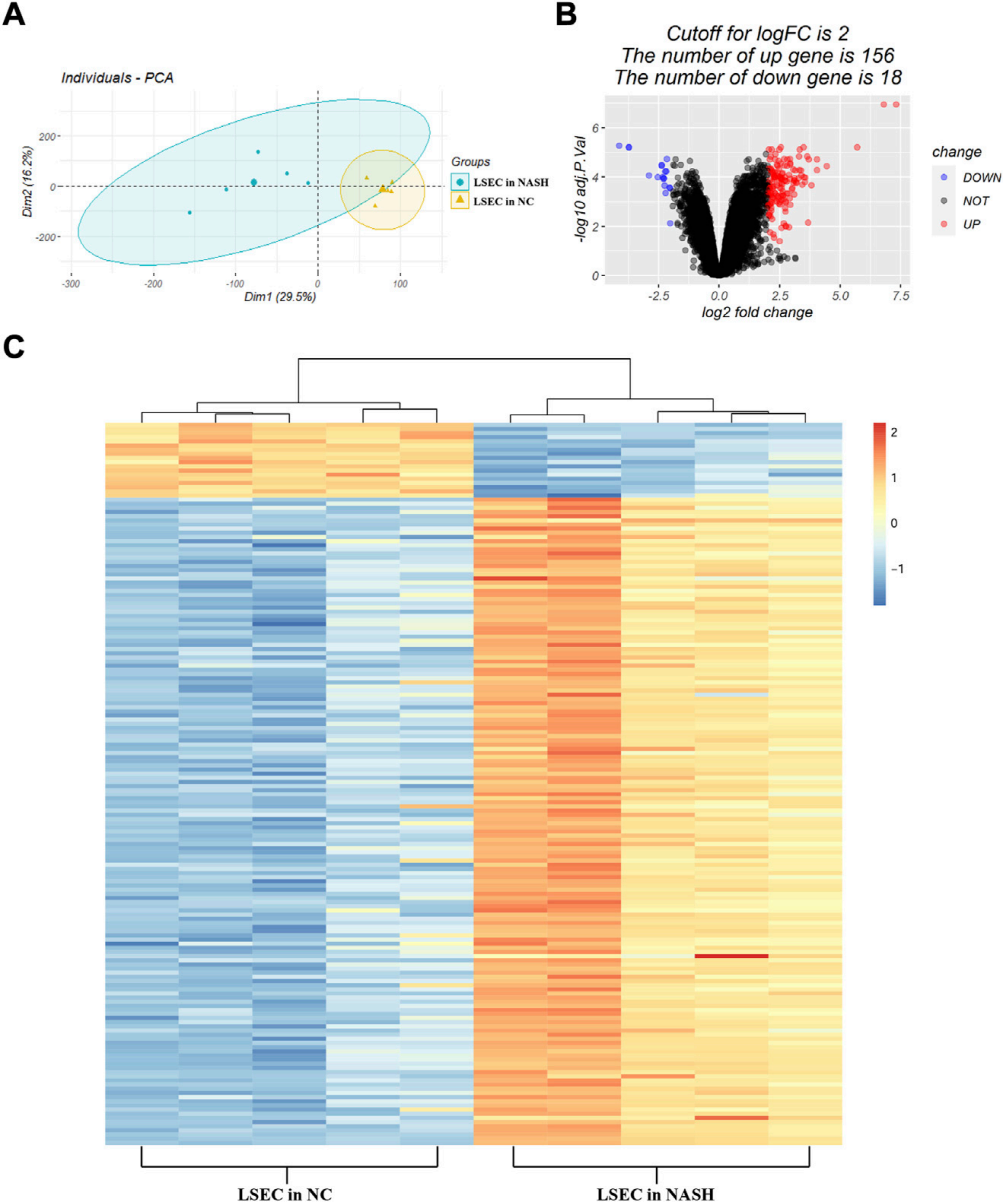
Identification of DEGs. **(A)** Principal component analysis between the normal samples and the NASH samples of LSECs; **(B)** volcano plot of DEGs between the normal samples and the NASH samples of LSECs. The red plots represent upregulated DEGs, the black plots represent non-significant genes, and the blue plots represent downregulated DEGs. **(C)** Heatmap of DEGs between the normal samples and the NASH samples of LSECs. Red rectangles represent high expression, and blue rectangles represent low expression.

Based on the analysis of the dataset GSE140994, a total of 174 DEGs were identified (listed in Supplementary Table S1). As shown in Figure 2A, the principal component analysis indicated a significant difference in the gene expression profile of LSECs between the NASH group and the normal control group. Among the 174 DEGs, there were 156 upregulated genes and 18 downregulated genes (Figure 2B). The heatmap plot was used to visualize these DEGs (Figure 1C).

### Identification of the tissue-/organ-specific expressed genes

A total of 84 tissue-/organ-specific expressed genes were identified according to BioGPS (Table 2). We observed that most of these genes were expressed in the immune organ/system (36/84, 42.86%). The digestive system was associated with the second most specifically expressed genes (17/84, 20.24%), followed by the others (10/84, 11.90%), the liver (8/84, 9.52%), the kidney (5/84, 5.95%), and the endocrine system (4/84, 4.76%). Finally, the nervous system and bone were associated with the fewest tissue-/organ-specific expressed genes (2/84, 2.38%) (Table 2).

**TABLE 2:** Distribution of tissue- and organ-specific expressed genes identified by BioGPS.

System/organ	Genes	Number
Immune organs/cells		
Lymph nodes/spleen	CXCL13, TIMD4, GBP2B, and CD209 B	4
Macrophage	PNP2, LY96, RAB7B, GADD45B, SLC7A2, CCL4, MYOF, IL1RN, ALCAM, PTGS2, ACOD1, FAM20C, SLAMF7, IL7R, ATF3, CCL3, RGS1, CLEC7A, CXCL2, CCL9, ATP6V0D2, and MMP12	22
Mast cell	PLXDC2 and CX3CR1	2
NK cell	OSBPL3	1
Multiple immune organs/cells	LTB, STAP1, H2-AB1, H2-AA, H2-EB1, LILR4B, and ITGAX	7
Bone	CD163 and SCARA3	2
Liver	C6, SERPINA1D, CRP, ITIH2, CFI, SERPINA1C, GC, and AMBP	8
Nervous system	RASGRF2 and MS4A7	2
Digestive system	CES2E, CLDN7, SPINT1, RETREG1, JCHAIN, KRT19, ANXA13, SERINC2, SLC5A1, KCNE3, CLDN3, CFTR, EPCAM, TSTD1, MMP7, IGHA, and KRT8	17
Endocrine system	CNTFR, CHST2, PALMD, and KCNN4	4
Kidney	HNF1B, CDH6, 1700011H14RIK, CLDN4, and PKHD1	5
Other	CES1D, CAPN6, PAMR1, DDR1, CLMN, OLFML3, TMEM45A, SFTPD, CP, and MME	10

Abbreviation: NK, natural killer.

### GO enrichment analysis results

**FIGURE 3:**
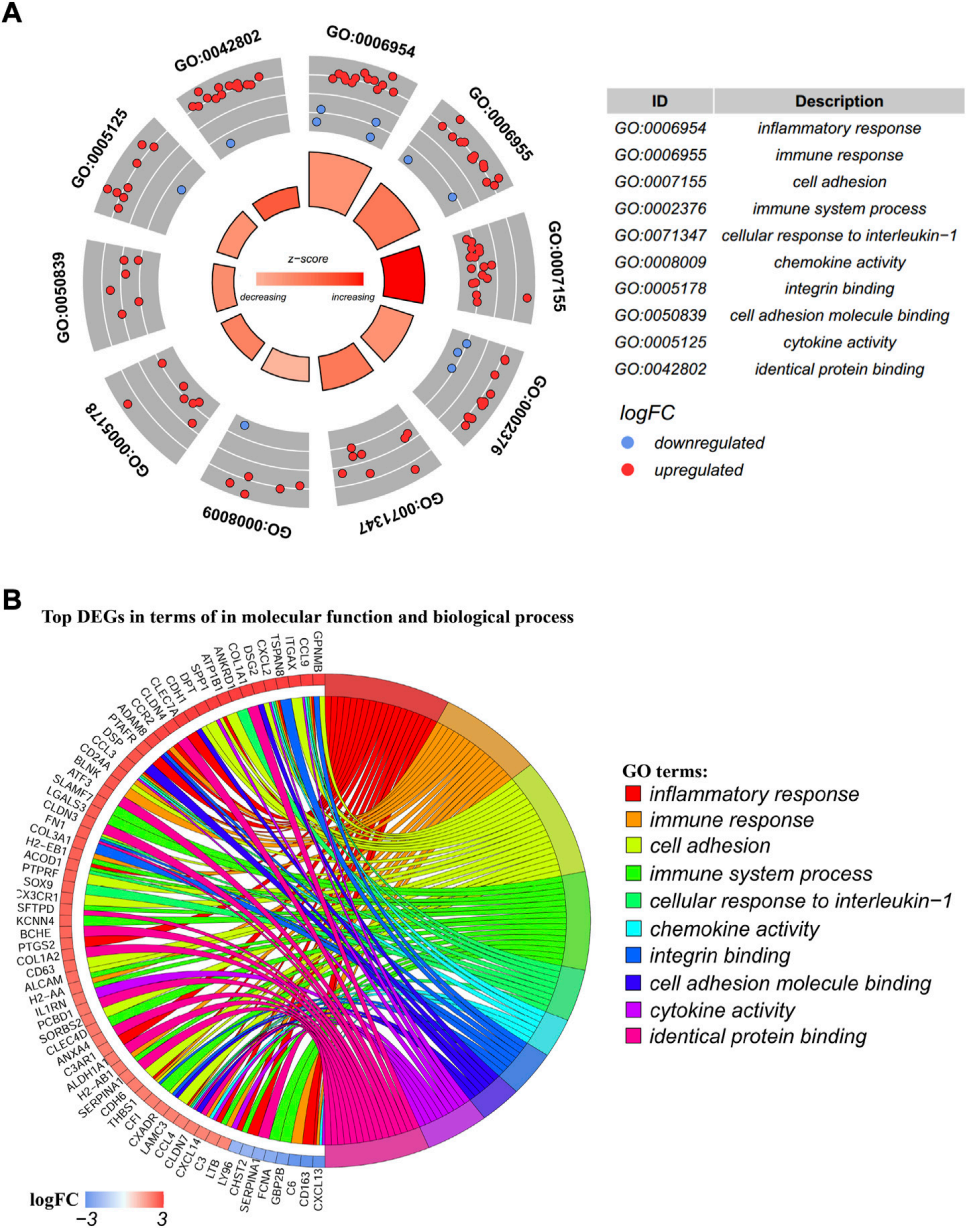
GO (Gene Ontology) terms enrichment analysis. **(A)** GO circle plot; the inner ring was a bar plot where the bar height showed the significance of the term (−log10 *p*-value) and the gradual color showed the z-score. The outer ring displayed scatter plots of the expression levels (logFC) for the genes in each term. The distribution of genes in different GO terms was used to predict different annotations. **(B)** GO chord plot of the relationship between the list of selected genes and their corresponding GO terms, together with the logFC of the genes. The left half of the GO chord displayed the up-expression or down-expression DEGs. The right half represented different GO terms with varied colors. A gene was linked to a certain GO term by the colored bands.

The biological process (BP) and molecular function (MF) of these 174 DEGs were analyzed by using the DAVID database. The ten most significantly enriched BP and MF terms were presented in the circle plot (Figure 3A). The outer circle is a scatter plot for each GO term with logFC assigned to genes. The red dots represented upregulated genes, and the blue dots represented downregulated genes. The 10 GO terms included the following: inflammatory response (GO:0006594), immune response (GO:0006955), cell adhesion (GO:0007155), immune system process (GO:0002376), cellular response to interleukin 1 (GO:0071347), chemokine activity (GO:0008009), integrin binding (GO:0005178), cell adhesion molecule binding (GO:0050839), cytokine activity (GO:0005125), and identical protein binding (GO:0042802). A chord plot displayed the distribution of each DEG to the corresponding pathway enrichment (Figure 3B).

### PPI network analysis, MCODE cluster modules, and hub gene identification

**FIGURE 4:**
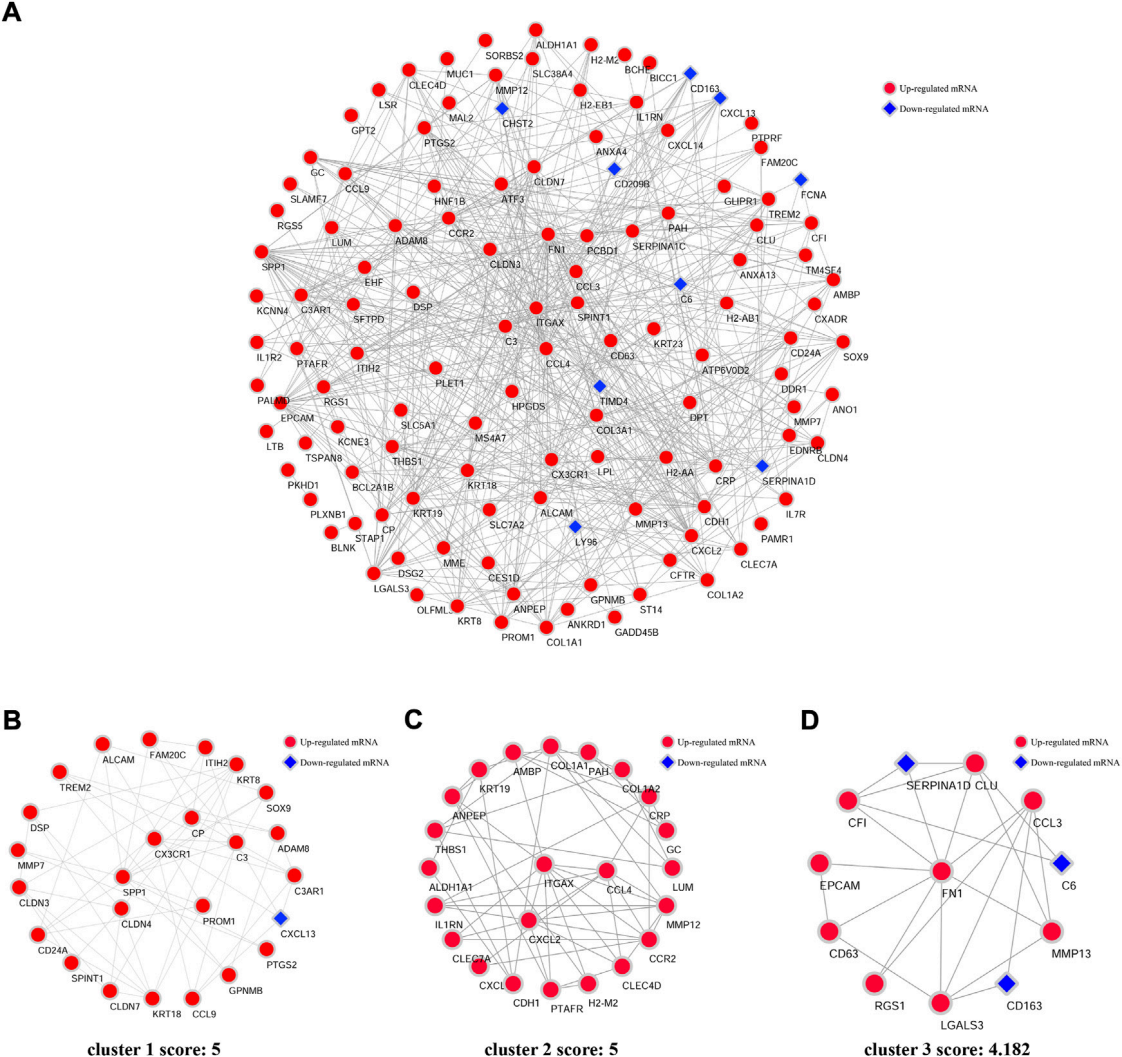
PPI network of DEGs and three clusters extracted by MCODE. **(A)** Using the Cytoscape software and online database STRING, the protein–protein interaction network coded by DEGs comprised 124 nodes and 526 edges. Each node represents a protein, while each edge represents the association of proteins. Red diamonds represent the upregulated DEGs, and blue hexagons represent the downregulated DEGs. Three cluster modules were extracted by MCODE. Cluster 1 **(B)** had high cluster score (score: 5, 25 nodes and 60 edges), followed by cluster 2 **(C)** (score: 5, 23 nodes and 55edges), and cluster 3 **(D)** (score: 4.182, 12 nodes and 23 edges).

An interaction network between the proteins coded by DEGs was predicted by STRING and visualized by Cytoscape (Figure 4A). The PPI network comprised 124 nodes and 526 edges, which indicated each DEG and the interaction between them, respectively. In this network, three cluster modules (Figures 4B–D) were identified by using the MCODE plugin according to the screening criteria. Module 1 (cluster score: 5; with 25 nodes and 60 edges) mainly contains DEGs involved in chemokine activity and expressed in the digestive system. The DEGs in module 2 (cluster score: 5; with 23 nodes and 55 edges) were mainly related to chemokine activity and expressed in the immune system. Module 3 (cluster score: 4.182; with 12 nodes and 23 edges) was mainly related to the signaling molecules of cytokines and chemokines.

Next, 15 hub genes including CCl4, FN1, CCR2, EPCAM, CDH1, CXCL2, CCL3, KRT19, SPP1, KRT18, C3, CX3CR1, KRT8, ITGAX, and CLDN3 were identified according to the five algorithms of the cytoHubba plugin. The detailed information on these 15 genes was listed in Table 3. As the immune reaction plays an important role in the pathogenesis of NASH and GO enrichment analyses showed that DEGs were mainly enriched in immune-activated and inflammatory pathways, we selected nine hub genes which are specifically expressed in the immune tissues/cells and the digestive system. These nine specifically expressed hub genes could be the most important genes in the PPI network.

**TABLE 3:** 15 hub genes identified by five algorithms of cytoHubba.

Hub genes	Description	log_2_FC	Regulation	Distribution
CCL4	Chemokine (C-C motif) ligand 4	2.099	Up	Macrophage
FN1	Fibronectin 1	2.599	Up	N/A
CCR2	Chemokine (C-C motif) receptor 2	2.902	Up	N/A
EPCAM	Epithelial cell adhesion molecule	3.172	Up	Digestive system
CDH1	Cadherin 1	3.069	Up	N/A
CXCL2	Chemokine (C-X-C motif) ligand 2	3.676	Up	Macrophage
CCL3	Chemokine (C-C motif) ligand 3	2.734	Up	Macrophage
KRT19	Keratin 19	2.254	Up	Digestive system
SPP1	Secreted phosphoprotein 1	3.097	Up	N/A
KRT18	Keratin 18	3.637	Up	N/A
C3	Complement component 3	2.053	Up	N/A
CX3CR1	Chemokine (C-X3-C motif) receptor 1	2.553	Up	Mast cell
KRT8	Keratin 8	3.48	Up	Digestive system
ITGAX	Integrin alpha X	4.03	Up	Multiple immune organs/cells
CLDN3	Claudin 3	2.608	Up	Digestive system

Abbreviation: N/A, not available.

### Prediction of mRNA–miRNA interaction and construction of the co-expressed network

**FIGURE 5:**
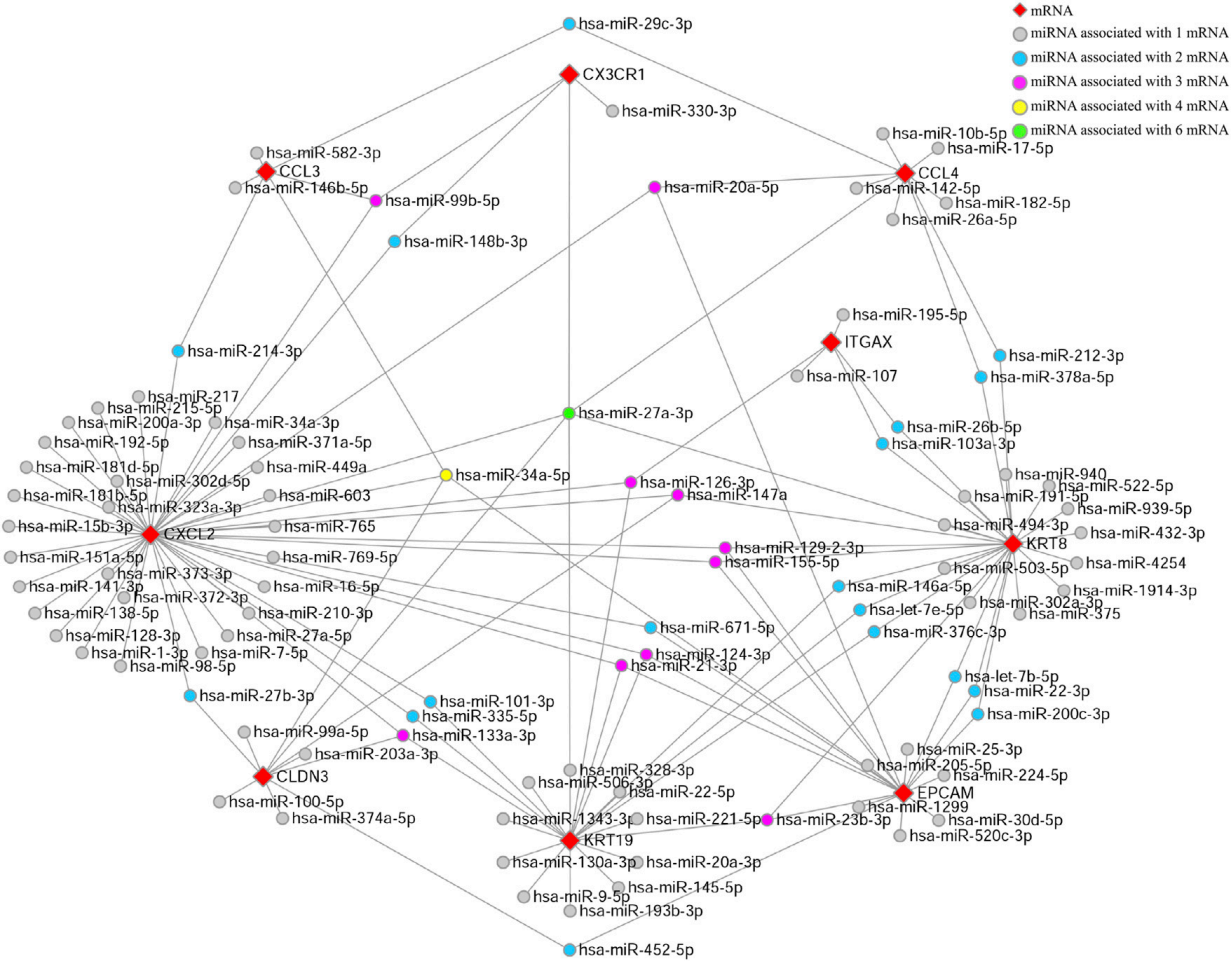
miRNA-mRNA-co-expressed network. A co-expressed network of mRNAs and target miRNAs. The mRNA–miRNA co-expressed network was constructed by Cytoscape including 107 nodes and 144 edges. Every node represents one miRNA/mRNA, and each edge represents the interaction of mRNA and miRNA. mRNAs and miRNAs are indicated with diamond and circle shapes, respectively.

We used the Network Analyst database to predict target miRNAs of hub genes. We obtained 98 target miRNAs for these nine specifically expressed hub genes and 144 mRNA–miRNA connections. A co-expressed network of mRNAs and miRNAs which comprised 107 nodes and 144 edges was plotted by Cytoscape (Figure 5). CXCL2 has the largest number of target miRNAs, while CX3CR1 has only four target miRNAs. Each diamond refers to a specific hub gene, while the spheres connected to the diamond refer to the target miRNAs. The colors of the spheres indicate a varying number of hub genes that are related to the miRNA. Thus, the miRNA hsa-miR-27a-3p with green color is related to the maximum number of hub genes, indicating that it might have the most complex and important biological functions.

### Verification of the nine specifically expressed hub genes

**FIGURE 6:**
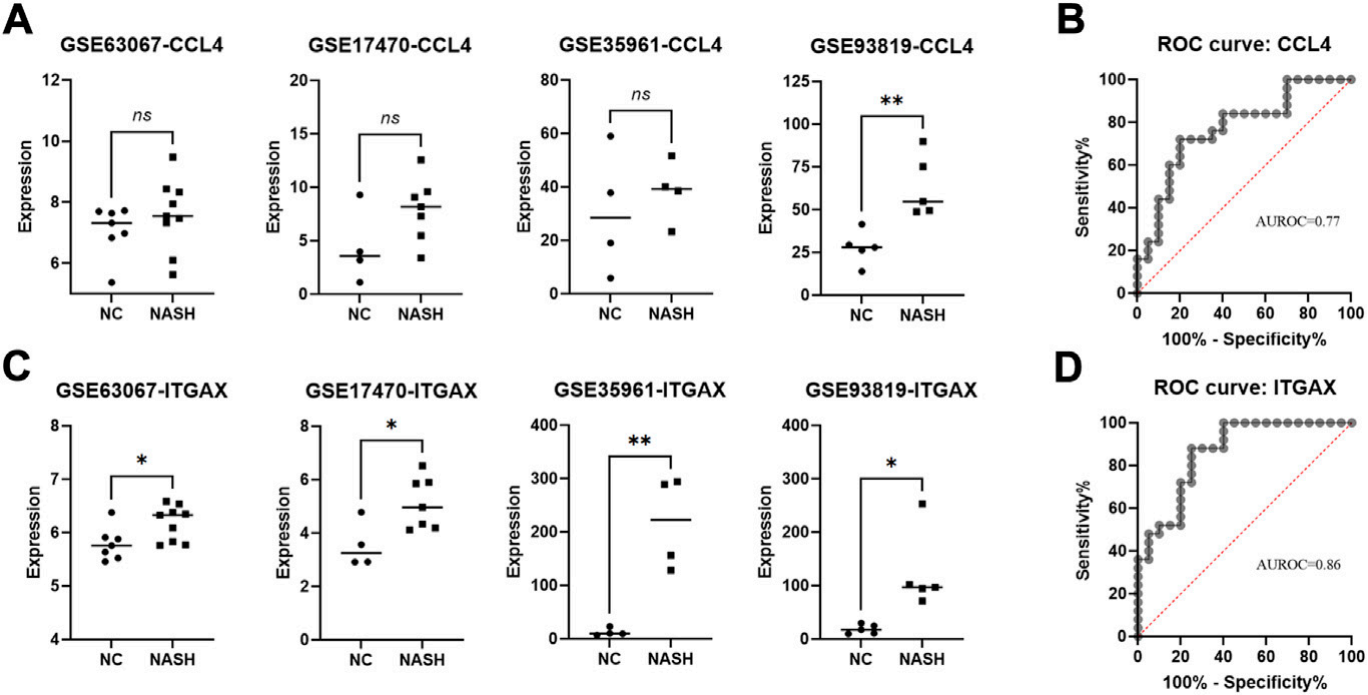
Identification of the CCL4 and ITGAX in four validation databases and investigation of its diagnosis value in NASH. **(A)** mRNA expression of CCL4 in NASH and normal samples was verified in four databases. **(B)** ROC curves of the CCL4 in the validating set. **(C)** mRNA expression of ITGAX in NASH and normal samples were verified in four databases. **(D)** ROC curves of ITGAX in the validating set. ns, non-significant; *p < 0.05 and ***p < 0.005 by *t*-test.

We further validated the expression of the nine specifically expressed hub genes in another four GEO databases as listed in Table 1. When examining the diagnostic performance for the hub genes, we included all the samples from these databases in a diagnostic analysis and calculated the area under the receiver operating characteristic (AUROC). As shown in Figure 6; Supplementary Figure S1, we found an increasing trend of CCL4 expression in the four databases (Figure 6A) with an AUROC value of 0.77 (Figure 6B). Meanwhile, the expression of ITGAX was significantly increased in the four databases (*p* < 0.05 for all, Figure 6C) with a favorable AUROC value of 0.86 (Figure 6D). On the other hand, for the other seven hub genes, no significantly increased/decreased expression (or trend) was identified in the four databases (Supplementary Figure S1).

### Verification of miRNA in the liver and serum of NASH patients

Using the dataset GSE59492, which contains the non-coding RNA profiling of the liver of NASH patients, we finally identified 37 out of 144 miRNAs that were downregulated in the liver of NASH patients (Table 4). Among these 37 miRNAs, there were 23 miRNAs that were also downregulated in the serum of NASH patients (Table 4). Among these miRNA, hsa-miR-378a, hsa-miR-17, and hsa-miR-20a are related to CCL4 in the mRNA–miRNA co-expression network (Figure 5), while hsa-miR-103a and hsa-miR-107 are related to ITGAX (Figure 5).

**TABLE 4:** Verification of miRNA in the liver and serum of NASH patients.

Downregulated miRNA in the liver of NASH patients	Fold change	Regulation in the serum of NASH patients	Fold change
hsa-miR-193b-3p	0.857	Up	1.925
hsa-miR-378a-5p	0.886	Down	0.346
hsa-miR-940	0.809	Down	0.768
hsa-miR-17-5p	0.945	Down	0.079
hsa-miR-20a-5p	0.943	Down	0.091
hsa-miR-20a-3p	0.943	Down	0.131
hsa-miR-603	0.785	N/A	N/A
hsa-miR-192-5p	0.961	Down	0.313
hsa-miR-210-3p	0.936	Down	0.161
hsa-miR-103a-3p	0.966	Down	0.101
hsa-miR-151a-5p	0.968	Down	0.095
hsa-miR-107	0.975	Down	0.247
hsa-miR-335-5p	0.900	Down	0.125
hsa-miR-101-3p	0.897	Down	0.086
hsa-miR-22-3p	0.981	Down	0.393
hsa-miR-22-5p	0.981	Down	0.393
hsa-miR-30d-5p	0.974	Down	0.285
hsa-miR-191-5p	0.981	Down	0.083
hsa-miR-494-3p	0.972	Down	0.283
hsa-miR-129-2-3p	0.924	Down	0.249
hsa-miR-582-3p	0.933	N/A	N/A
hsa-miR-215-5p	0.973	N/A	N/A
hsa-miR-16-5p	0.991	Down	0.189
hsa-miR-217	0.951	N/A	N/A
hsa-miR-372-3p	0.948	N/A	N/A
hsa-miR-133a-3p	0.966	Down	0.19
hsa-miR-302a-3p	0.964	Down	0.111
hsa-let-7b-5p	0.995	Down	0.126
hsa-miR-147a	0.968	N/A	N/A
hsa-miR-203a-3p	0.983	N/A	N/A
hsa-miR-374a-5p	0.971	N/A	N/A
hsa-miR-506-3p	0.985	N/A	N/A
hsa-miR-124-3p	0.988	N/A	N/A
hsa-miR-4254	0.988	N/A	N/A
hsa-miR-23b-3p	0.999	Down	0.116
hsa-miR-1343-3p	0.998	N/A	N/A
hsa-miR-302d-5p	0.999	N/A	N/A

Abbreviation: N/A, not available.

### Prediction of target non-coding RNAs and ceRNA networks

**FIGURE 7:**
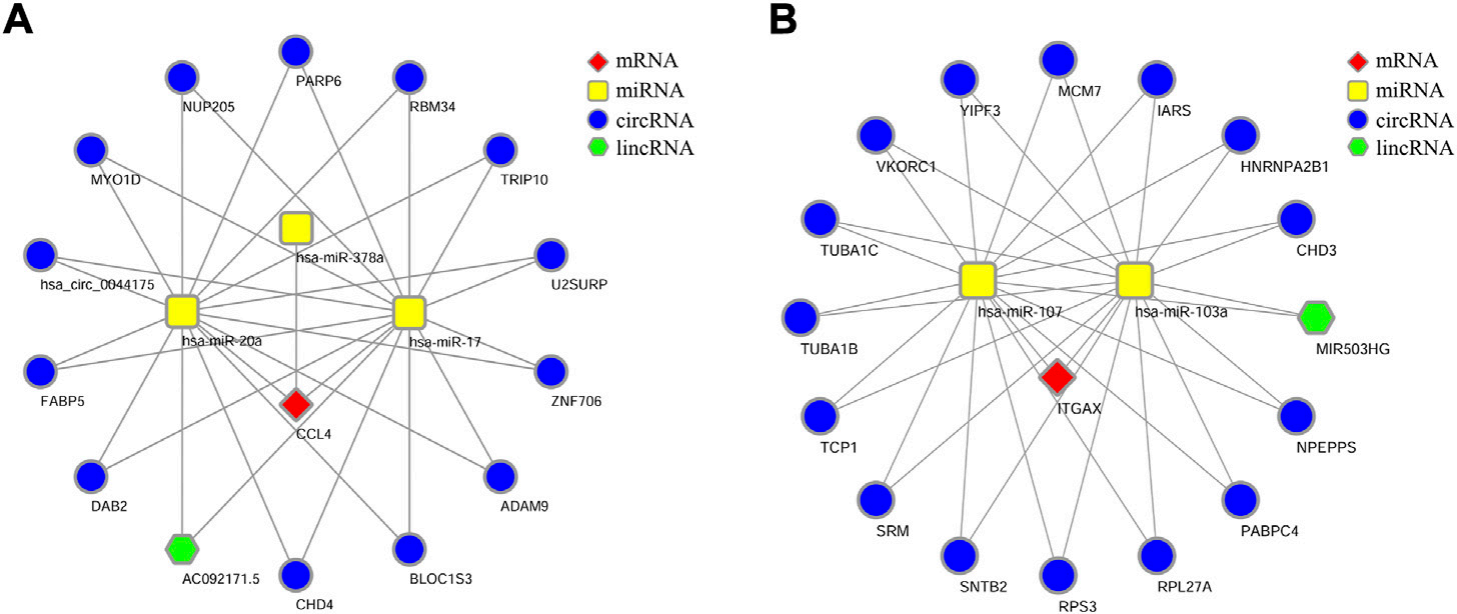
lncRNA/circRNA-miRNA-mRNA-ceRNA network. **(A)** Network of CCL4-IncRNA-miRNA-mRNA. **(B)** Network of ITGAX-IncRNA-miRNA-mRNA.

MiRNAs are known to induce gene silencing and downregulate gene expression by binding to mRNAs. However, its upstream molecules, such as circRNAs and lncRNAs, can affect the function of miRNA by combining miRNA response elements, thus regulating gene expression *via* the transcript process. This interaction between RNAs is called a ceRNA network. Subsequently, we explored the lncRNAs and circRNAs related to the aforementioned miRNAs by using the Starbase tool (https://starbase.sysu.edu.cn/). Finally, we obtained the networks involving 13 circRNAs, one lncRNA related to the miRNAs of CCL4 (Figure 7A), 15 circRNAs, and one lncRNA related to the miRNAs of ITGAX (Figure 7B). We propose that the lincRNA-circRNA-miRNA-CCL4 and lincRNA-circRNA-miRNA-ITGAX networks might be potential key pathways to regulate the disease of NASH.

## Discussion

In our study, we obtained 174 DEGs of LSECs in NASH. GO enrichment analysis revealed in transcript levels that these DEGs mainly participated in the inflammatory response and cell adhesion activity in NASH. GO pathways that were enriched included inflammatory and immunological pathways involving inflammatory response interaction, cell adhesion, cellular response to the interleukin-1 signaling pathway, and chemokine and cytokine activities. These results suggest that LSECs in NASH have distinct immune activation which is one of the main causes of liver inflammation and fibrosis.

Two immune system-specifically expressed genes, namely, CCL4 and ITGAX, were finally identified. CCL4 and ITGAX were both upregulated in LSECs in NASH with statistical significance (*p* < 0.05). ROC curves showed that both genes had a certain diagnostic value for both human and animal NASH. CCL4 and ITGAX can be potential biomarkers for early diagnosis of NASH. In addition, we constructed an mRNA–miRNA co-expression network and ceRNA networks to further understand the pathogenesis of NASH at the transcriptome level. The prediction of upstream miRNAs, lncRNAs, and circRNAs led to the identification of a potential mRNA-miRNA-lncRNA/circRNA regulatory network.

ITGAX (integrin alpha x), also known as CD11c, a member of the integrin family, serves as a receptor for the extracellular matrix. ITGAX is a surface marker that can be expressed on dendritic cells, B lymphocytes, T lymphocytes, NK cells, and subsets of monocytes and macrophages (Poltorak and Schraml, 2015). The expression of ITGAX by LSECs has been reported in the previous publication (Knolle et al., 1999). Flow cytometry showed that LSECs expressed ITGAX *in vitro*, which could be downregulated by treatment of cells with endotoxin (Knolle et al., 1999). This could explain that *in situ* studies showed the lack of ITGAX expression on LSECs because LSECs are physiologically exposed to endotoxin in portal venous blood. Therefore, under certain circumstances, such as *in vitro* or hepatopathy, the expression of ITGAX by LSECs might be activated. Recently, accumulating evidence indicates that ITGAX might play an important role in cell death, cytokine processing, and inflammation (Wang et al., 2019). An increased number of ITGAX^+^ cells has been found in the liver of mice under obese conditions (Stefanovic-Racic et al., 2012), which is one of the key triggers for liver inflammation progression from NAFLD to NASH (Younossi et al., 2016). This indicates that ITGAX might be a significant mediator in the pathogenesis of NASH. Our study found that ITGAX was significantly upregulated in the LSECs in NASH. The ROC curve of ITGAX indicated that it had a very strong correlation with NASH (AUROC = 0.86). ITGAX is considered a promising biomarker for the diagnosis of NASH.

CCL4, also known as macrophage inflammatory protein-1β, is a chemo-attractant for varieties of immune cells (Zhao et al., 2021). CCR5, the receptor of CCL4, is expressed by various cells, including T lymphocytes, NK, NKT cells, monocytes, macrophages, and γδ T cells. Quite a few previous reports as well as our previous article have proved that LSEC is capable of expressing inflammatory mediators including cytokines and chemokines (Wang et al., 2021). CCL4 has also been found to be expressed in vascular endothelial cells (Barker et al., 2008). It is found that inflammatory factors, such as IL-1beta, can mediate the upregulation of CCL4 production in the hepatic cells, thus contributing to the continuous recruitment of activating immunocytes to the liver (Zhang et al., 2003). The increased CCL4 is related to critical biological processes of inflammation in the liver. In addition, miRNAs could upregulate CCL4 expression in immune cells (Cheng et al., 2015). In our study, we also found several circRNAs and lncRNAs that were related to the regulation of CCL4 expression in LSECs. Therefore, further investigation might as well focus on illustrating the mechanism of the CCL4-mRNA-miRNA-lncRNA/circRNA regulatory network in NASH. LncRNAs have emerged as significant factors in almost all processes of gene function and regulation. In addition, it has been reported that the Mir503HG is involved in tumor metastasis in hepatocellular carcinoma (Wang et al., 2018). Therefore, MIR503HG-hsa-miR-107/hsa-miR-103a/-ITGAX might be a potential ceRNA regulatory pathway to regulate the disease progression of NASH.

There are limitations to this study. The major disadvantage of the MCD diet model is the absence of insulin resistance and metabolic syndrome. In an MCD diet model, loss of body weight is the converse of obesity-related NASH. Meanwhile, in the MCD diet model, initial fatty changes can be observed in the mice liver after 2 weeks of MCD diet followed by progressive deterioration to evident NASH 6 weeks after the beginning of treatment. Thus, initial simple steatosis can progress into NASH within 6 weeks in the MCD diet model. The liver pathology examination also revealed that the MCD diet model recapitulates the major characteristics of human NASH, including steatosis, ballooning degeneration, inflammation, and fibrosis. Therefore, the MCD diet model performs well in mimicking the changes in the intrahepatic environment, especially the liver-specific cells such as LSECs. It would be better that the changes of LSECs can be further validated in other models such as high-fat diet-induced NAFLD/NASH.

## Conclusion

In this study, we systematically analyzed the differentially expressed genes of LSECs in NASH. We identified two immune system-specifically expressed genes, CCL4 and ITAGX, expressed by LSECs, as potential key players for the pathogenesis of disease development in NASH at the transcriptome level. Our findings put forward promising therapeutic and prognostic values of the genes expressed by LSECs as well as the ceRNA regulatory networks in NASH.

## Data Availability

The original contributions presented in the study are included in the article/Supplementary Material; further inquiries can be directed to the corresponding authors.

## Abbreviations

NASH - nonalcoholic steatohepatitis; NAFLD - nonalcoholic fatty liver disease; LSECs - liver sinusoidal endothelial cells; NK - natural killer; LPS - lipopolysaccharide; ceRNA - competitive endogenous RNA; MCD - methionine–choline-deficient diet; ALT - alanine aminotransferase; AST - aspartate aminotransferase; SEM - scanning electron microscopy; TEM - transmission electron microscopy; GO - Gene Ontology; DEG - differentially expressed genes; PPI - protein–protein interaction; MCODE - Minimal Common Oncology Data Elements; BP - biological process; MF - molecular function; ITGAX - integrin alpha x.
