# Supplementary material for: Signature of gene expression profile of liver sinusoidal endothelial cells in nonalcoholic steatohepatitis
Source: Front Cell Dev Biol. 2022 Sep 21;10:946566. doi: 10.3389/fcell.2022.946566 (PMC9533023; doi:10.3389/fcell.2022.946566)
Supplement: Supplementary file 1 [file Table1.docx]

**Table S1. Information of 174 DEGs**

| DEG | logFC | Adjusted P value |
| --- | --- | --- |
| CXCL13 | -4.13676 | 5.23E-06 |
| TIMD4 | -3.73293 | 6.20E-06 |
| CD163 | -3.72797 | 6.20E-06 |
| C6 | -2.89806 | 8.70E-05 |
| GBP2B | -2.54201 | 9.89E-05 |
| GM19461 | -2.37617 | 3.12E-05 |
| CNTFR | -2.37332 | 3.51E-05 |
| CD209B | -2.34141 | 0.0001 |
| PNP2 | -2.30449 | 0.000115 |
| FCNA | -2.28968 | 0.00011 |
| RASGRF2 | -2.25155 | 0.000218 |
| DUSP4 | -2.2336 | 5.79E-05 |
| SERPINA1D | -2.21523 | 0.000447 |
| CES2E | -2.19474 | 5.79E-05 |
| CYP2C69 | -2.16526 | 1.84E-05 |
| CHST2 | -2.0528 | 0.000264 |
| OLFR1396 | -2.02831 | 0.000285 |
| LY96 | -2.02541 | 0.007458 |
| PLXDC2 | 2.000206 | 0.001612 |
| PDZK1IP1 | 2.014456 | 0.000447 |
| SNORD72 | 2.020729 | 0.001344 |
| LTB | 2.022079 | 0.00049 |
| HPGDS | 2.029506 | 0.001397 |
| EDNRB | 2.045312 | 4.40E-05 |
| OSBPL3 | 2.046879 | 7.55E-06 |
| RAB7B | 2.052728 | 0.003457 |
| C3 | 2.053142 | 0.000173 |
| RNU3A | 2.054757 | 0.000538 |
| GADD45B | 2.056719 | 0.003612 |
| CRP | 2.060935 | 0.000553 |
| ITIH2 | 2.066617 | 0.000218 |
| CXCL14 | 2.072276 | 0.002891 |
| SLC7A2 | 2.072287 | 0.000297 |
| PALMD | 2.075415 | 0.000135 |
| ANO1 | 2.078124 | 0.000272 |
| CLU | 2.083168 | 0.000276 |
| CLDN7 | 2.09162 | 1.88E-05 |
| CCL4 | 2.099417 | 0.017503 |
| IGKC | 2.105822 | 0.001204 |
| MAL2 | 2.110557 | 4.77E-05 |
| LAMC3 | 2.117982 | 0.00126 |
| STAP1 | 2.119646 | 0.00393 |
| SPINT1 | 2.119931 | 0.000425 |
| CXADR | 2.129883 | 4.44E-05 |
| HNF1B | 2.129885 | 0.000526 |
| WFDC15B | 2.13294 | 1.43E-05 |
| CFI | 2.138093 | 7.26E-05 |
| 5330417C22RIK | 2.138283 | 0.001006 |
| CES1D | 2.155861 | 0.002294 |
| THBS1 | 2.156247 | 0.000447 |
| CAPN6 | 2.159867 | 0.001732 |
| CDH6 | 2.172067 | 7.55E-06 |
| RETREG1 | 2.181634 | 0.001079 |
| JCHAIN | 2.186239 | 0.005087 |
| SERPINA1C | 2.188646 | 0.000157 |
| H2-AB1 | 2.189803 | 5.37E-05 |
| ALDH1A1 | 2.193639 | 6.40E-05 |
| C3AR1 | 2.196897 | 0.000711 |
| SNORD45B | 2.197078 | 0.00063 |
| ANXA4 | 2.205697 | 6.40E-05 |
| MYOF | 2.208208 | 0.000292 |
| PLXNB1 | 2.213491 | 0.000251 |
| CLEC4D | 2.241428 | 0.021108 |
| PAMR1 | 2.251178 | 0.000238 |
| KRT19 | 2.254012 | 8.20E-05 |
| IGKV1-110 | 2.258708 | 0.028543 |
| SORBS2 | 2.262319 | 0.0001 |
| EMP1 | 2.271089 | 0.00012 |
| PCBD1 | 2.274025 | 6.40E-05 |
| SLAMF9 | 2.275187 | 9.64E-05 |
| IL1RN | 2.277371 | 0.000741 |
| DDR1 | 2.279514 | 9.11E-05 |
| CLMN | 2.286619 | 0.000425 |
| MYO1B | 2.297645 | 0.000103 |
| SLC39A4 | 2.305064 | 1.88E-05 |
| DCDC2A | 2.316016 | 1.17E-05 |
| PROM1 | 2.323729 | 0.000456 |
| H2-AA | 2.354273 | 8.20E-05 |
| SNORA75 | 2.360362 | 0.000555 |
| MMP13 | 2.369458 | 0.000954 |
| 1700011H14RIK | 2.38058 | 3.12E-05 |
| ANXA13 | 2.382812 | 0.000212 |
| ALCAM | 2.406664 | 9.49E-05 |
| LSR | 2.409584 | 3.45E-05 |
| SERINC2 | 2.417791 | 6.33E-05 |
| SNORA81 | 2.425048 | 0.000594 |
| SLC5A1 | 2.433505 | 0.000731 |
| RGS5 | 2.45216 | 2.22E-05 |
| CD63 | 2.463854 | 4.55E-05 |
| COL1A2 | 2.470324 | 0.001175 |
| KCNE3 | 2.474037 | 7.55E-06 |
| RNF128 | 2.478925 | 0.000173 |
| IL1R2 | 2.497774 | 0.040015 |
| OLFML3 | 2.499432 | 0.000272 |
| LUM | 2.500984 | 0.001268 |
| SLC38A4 | 2.502729 | 9.64E-05 |
| PTGS2 | 2.509394 | 0.001006 |
| BCHE | 2.522849 | 9.89E-05 |
| KCNN4 | 2.529885 | 0.00011 |
| TMEM45A | 2.530203 | 2.79E-05 |
| SFTPD | 2.550979 | 6.20E-06 |
| CX3CR1 | 2.552731 | 0.000601 |
| SOX9 | 2.556655 | 0.000843 |
| PTPRF | 2.561939 | 0.000124 |
| GPT2 | 2.570508 | 0.000114 |
| ACOD1 | 2.571015 | 0.011498 |
| H2-M2 | 2.577749 | 0.000173 |
| H2-EB1 | 2.579313 | 0.000279 |
| KRT23 | 2.586945 | 0.000103 |
| COL3A1 | 2.592239 | 0.000493 |
| PAH | 2.597348 | 0.000195 |
| FN1 | 2.599058 | 7.42E-05 |
| CLDN3 | 2.608821 | 6.80E-05 |
| FAM20C | 2.616592 | 0.000371 |
| LGALS3 | 2.620509 | 0.000565 |
| SLAMF7 | 2.653537 | 0.000787 |
| IL7R | 2.653637 | 0.000574 |
| ATF3 | 2.671116 | 0.007519 |
| GC | 2.684412 | 1.81E-05 |
| PLET1 | 2.694759 | 3.32E-05 |
| BLNK | 2.709674 | 0.000135 |
| AMBP | 2.717421 | 0.000138 |
| SCARA3 | 2.718788 | 9.89E-05 |
| CD24A | 2.724821 | 0.001818 |
| CCL3 | 2.733821 | 0.002506 |
| LILR4B | 2.739088 | 0.009606 |
| DSP | 2.741512 | 0.000971 |
| PTAFR | 2.745331 | 0.00107 |
| IGHV4-1 | 2.758432 | 0.010002 |
| LPL | 2.789321 | 0.00015 |
| SLC44A3 | 2.79152 | 0.000456 |
| CP | 2.806251 | 3.45E-05 |
| CFTR | 2.815654 | 1.88E-05 |
| SNORD89 | 2.839107 | 0.000464 |
| BICC1 | 2.876396 | 8.20E-05 |
| ADAM8 | 2.885377 | 0.010728 |
| CCR2 | 2.902353 | 0.000726 |
| CLDN4 | 2.916998 | 1.81E-05 |
| PKHD1 | 2.926939 | 0.000115 |
| EHF | 2.931438 | 2.73E-05 |
| ST14 | 2.935768 | 2.94E-05 |
| RGS1 | 2.937749 | 0.001339 |
| MS4A7 | 2.996112 | 0.00031 |
| GLIPR1 | 3.019751 | 0.000276 |
| CLEC7A | 3.051627 | 4.77E-05 |
| CDH1 | 3.068601 | 0.000167 |
| DPT | 3.089483 | 0.000497 |
| SNORD123 | 3.093585 | 0.000136 |
| SPP1 | 3.097236 | 0.000137 |
| ANPEP | 3.103358 | 0.000109 |
| MME | 3.134792 | 5.37E-05 |
| EPCAM | 3.171724 | 0.00016 |
| MUC1 | 3.195738 | 0.000176 |
| TM4SF4 | 3.283987 | 0.00011 |
| TSTD1 | 3.293992 | 1.43E-05 |
| ATP1B1 | 3.311347 | 5.11E-05 |
| ANKRD1 | 3.323848 | 7.55E-06 |
| MMP7 | 3.380289 | 5.37E-05 |
| IGHA | 3.395567 | 8.72E-05 |
| COL1A1 | 3.434812 | 0.000203 |
| KRT8 | 3.480136 | 6.20E-06 |
| LURAP1L | 3.50316 | 0.000102 |
| BCL2A1B | 3.532428 | 0.00016 |
| DSG2 | 3.553558 | 0.000104 |
| KRT18 | 3.637342 | 1.43E-05 |
| CXCL2 | 3.675991 | 0.006955 |
| TREM2 | 3.702026 | 1.88E-05 |
| IGKV1-88 | 3.811056 | 0.000211 |
| TSPAN8 | 4.028864 | 1.88E-05 |
| ITGAX | 4.029806 | 5.11E-05 |
| CCL9 | 4.439703 | 3.53E-05 |
| ATP6V0D2 | 5.704251 | 6.20E-06 |
| MMP12 | 6.795079 | 1.10E-07 |
| GPNMB | 7.307194 | 1.10E-07 |

Abbreviation: DEG, diferentially expressed gene; FC, fold change.
