# Supplementary figures and images for: Signature of gene expression profile of liver sinusoidal endothelial cells in nonalcoholic steatohepatitis
Source: Front Cell Dev Biol. 2022 Sep 21;10:946566. doi: 10.3389/fcell.2022.946566 (PMC9533023; doi:10.3389/fcell.2022.946566)

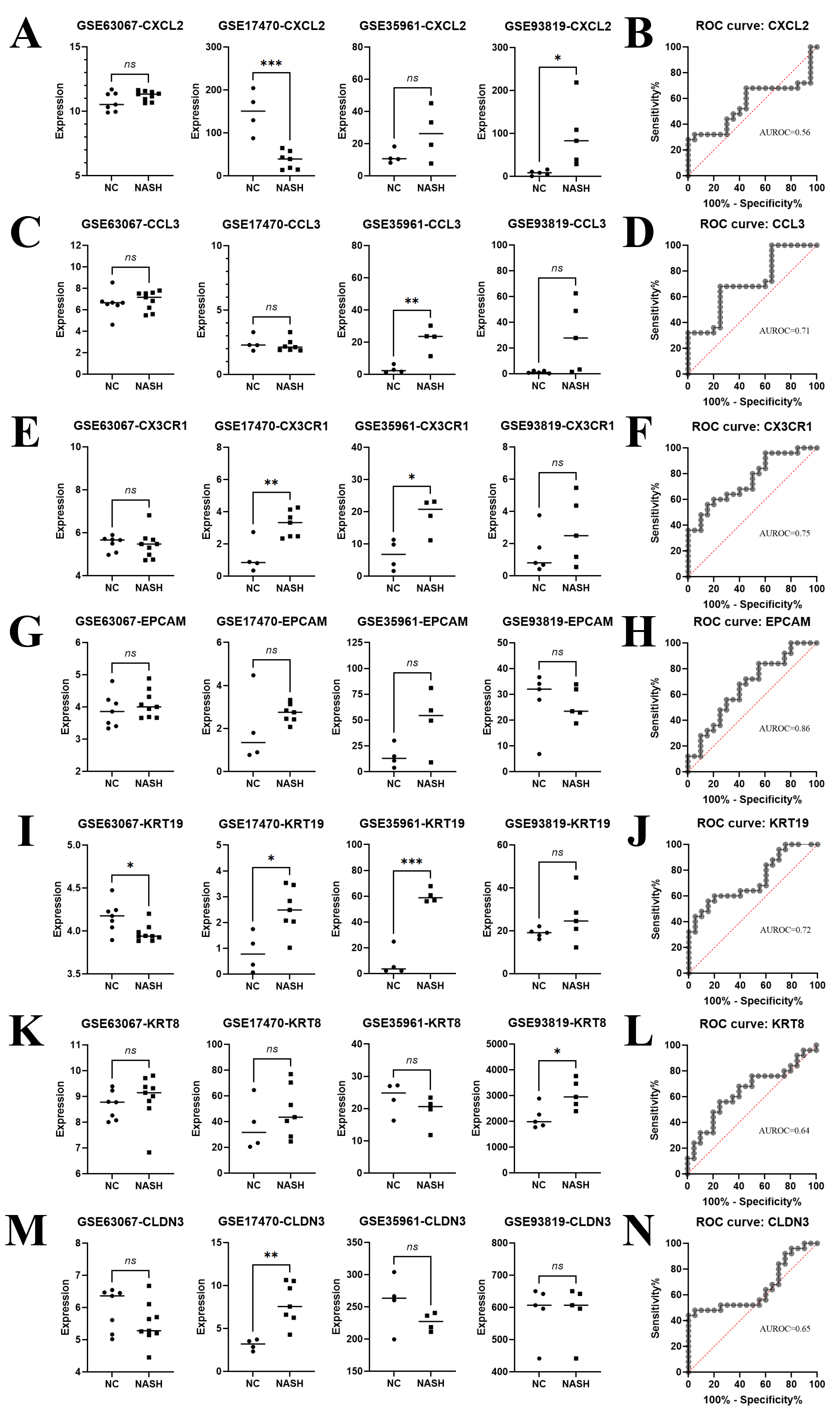

Supplement: Supplementary file 2 [file Image1.TIF]
